# Palmitate Inhibits Mouse Macrophage Efferocytosis by Activating an mTORC1-Regulated Rho Kinase 1 Pathway: Therapeutic Implications for the Treatment of Obesity

**DOI:** 10.3390/cells11213502

**Published:** 2022-11-04

**Authors:** László Sós, Éva Garabuczi, Tibor Sághy, Gábor Mocsár, Zsuzsa Szondy

**Affiliations:** 1 Doctoral School of Dental Sciences, Faculty of Dentistry, University of Debrecen, 4032 Debrecen, Hungary; 2 Department of Integrative Health Sciences, Institute of Health Sciences, Faculty of Health Sciences, University of Debrecen, 4032 Debrecen, Hungary; 3 Department of Biophysics and Cell Biology, Faculty of Medicine, University of Debrecen, 4032 Debrecen, Hungary; 4 Section of Dental Biochemistry, Department of Basic Medical Sciences, Faculty of Dentistry, University of Debrecen, 4032 Debrecen, Hungary; 5 Department of Biochemistry and Molecular Biology, Faculty of Medicine, University of Debrecen, 4032 Debrecen, Hungary

**Keywords:** palmitate, macrophage, efferocytosis, AMPK, mTORC1, ROCK1, autophagy, chronic inflammation, obesity

## Abstract

Every day, billions of our cells die and get cleared without inducing inflammation. When, clearance is improper, uncleared cells undergo secondary necrosis and trigger inflammation. In addition, proper efferocytosis would be required for inducing resolution of inflammation, thus clearance deficiencies in the long term lead to development of various chronic inflammatory diseases. Increasing evidence indicates that obesity, itself being a low-grade inflammatory disease, predisposes to a variety of other chronic inflammatory diseases. Previous studies indicated that this later might be partially related to an impaired efferocytosis induced by increased uptake of circulating saturated fatty acids by macrophages in obese people. Here, we show that palmitate inhibits efferocytosis by bone marrow-derived macrophages in a dose-dependent manner. Palmitate triggers autophagy but also activates an energy-sensing mTORC1/ROCK1 signaling pathway, which interferes with the autophagosome–lysosome fusion, resulting in accumulation of the cellular membranes in autophagosomes. We propose that lack of sufficient plasma membrane supply attenuates efferocytosis of palmitate-exposed macrophages. AMP-activated protein kinase activators lead to mTORC1 inhibition and, consequently, released the palmitate-induced efferocytosis block in macrophages. Thus, they might be useful in the treatment of obesity not only by affecting metabolism thought so far. ROCK1 inhibitors could also be considered.

## 1. Introduction

Timed induction of apoptosis and efficient phagocytosis of apoptotic cells by macrophages plays a determining role in maintaining tissue homeostasis. Every day, billions of our cells die and get cleared without triggering inflammation or autoimmunity [1]. Efferocytosis is mediated via various phagocytic receptors on macrophages out of which many recognize directly, or indirectly (via bridging molecules), phosphatidylserine that appears on the surface of apoptotic cells [2]. Macrophages in different tissues express different groups of these receptors, but all of them accumulate and function together in the phagocytic synapse to participate in the tethering and to trigger sufficient engulfment signaling once they interact with the cell surface molecules of the apoptotic cells [3]. Mouse macrophages are capable of forming two such engulfment portals simultaneously, and once these are generated, they will be used for the continuous uptake of dying cells as long as dying cells are around [4]. So far, two parallel evolutionarily conserved efferocytic signaling pathways have been shown to be induced by these phagocytic receptors, and both lead to the activation of the small GTPase Rac1 [5]. In addition to Rac1, other small GTPases, such as RhoA and Cdc42, have also been shown to contribute to the efferocytosis process. These G proteins work in a temporally regulated manner, in which Rac1 and Cdc42 act first together to initiate phagocytic cup formation via inducing actin polymerization, followed by RhoA activation which induces retraction of the phagocytic cup into the macrophage and subsequent phagosome internalization [5]. However, prolonged stimulation of RhoA has been shown to inhibit efferocytosis by macrophages via activating the Rho-associated protein kinase (ROCK) [6].

For proper functioning of the efferocytic signaling pathway, phosphoinositide-3 kinases (PI3Ks) are also activated to generate 3-phosphoinositides in the inner leaflet of the plasma membrane, which guide the recruitment of guanine exchange factors (GEFs) of the above-mentioned G proteins into the phagocytic cup [7,8,9], as well as the phagosome maturation at later phases of the engulfment process. Phagosome maturation is required for proper phagosome–lysosome fusion followed by apoptotic cell degradation mediated by the lysosomal enzymes [10]. Interestingly, some early PI3K-dependent steps of the autophagy process and the noncanonical form of efferocytosis seem to overlap, as after phagosome sealing, LC3-family proteins are conjugated to lipids at the phagosomal membrane by certain members of the canonical autophagy machinery (such as beclin-1, autophagy related genes (atg) 5 and 7) [11,12] to promote efficient phagosome–lysosome fusion, and inhibition of their activity or silencing their expression inhibit both processes. However, the process of LC3-dependent efferocytosis and autophagy is different, and while autophagy is controlled by mammalian target of rapamycin (mTOR)C1 activity [13], LC3-associated phagocytosis is not [12].

Apoptotic cell uptake, on one hand, requires massive cytoskeletal remodeling; on the other hand, macrophages are simultaneously facing the need of providing enough plasma membrane to both fully seal the apoptotic cells and to maintain the cell-surface membrane area [14]. This is achieved via endosomal [15] and lysosomal [16] membrane trafficking to the cell surface mediated by PI3Ks and synaptotagmin VII, respectively. The RAB family of GTPases also participates in the process, as RAB17 distributes phagolysosomal membranes into recycling endosomes, which then fuse to the cell membrane [17]. Rab17-mediated recycling endosomes and plasma membranes, however, also contribute to the autophagosome formation [18,19], indicating an overlapping pool of membranes promoting both efferocytosis and autophagy.

Previous studies have demonstrated that in obesity, the levels of circulating saturated fatty acids are high, and increased uptake of saturated fatty acids, such as palmitate, by macrophages leads to impaired clearance of apoptotic cells [20]. Since impaired clearance of apoptotic cells is linked to the development of various chronic inflammatory diseases [2], it might contribute to the high prevalence of chronic inflammatory diseases associated with obesity [21,22,23]. The aim of the present studies was to investigate the mechanism by which palmitate uptake interferes with the apoptotic cell clearance to identify drug targets to prevent it.

## 2. Materials and Methods

### 2.1. Reagents

All reagents were obtained from Sigma-Aldrich (Budapest, Hungary) except when indicated otherwise.

### 2.2. Animals

The experiments were carried out with bone marrow-derived macrophages (BMDMs) differentiated from the bone marrow of 2- to 4-month-old C57BL/6 male mice. Mice were bred and maintained under specific pathogen-free conditions in the Central Animal Facility, University of Debrecen. All animal experiments were approved by the Animal Care and Use Committee of the University of Debrecen (DEMÁB) with a permission number 7/2016/DEMÁB and 7/2021/DEMÁB.

### 2.3. Generation of BMDMs and Treatments

Bone marrow progenitors were obtained from the femur of 2- to 4-month-old mice after being sacrificed by isoflurane overdose. BMDMs were generated as we described previously [24]. At day 6, they were exposed for 24 h to bovine serum albumin (BSA) or to various concentrations of BSA-conjugated palmitate (prepared as it was described [25]) alone or together with 0.5 mM 5-aminoimidazole-4-carboxamide-1-β-D-ribofuranoside (AICAR), an AMP-activated protein kinase (AMPK) activator [26], 100 nM rapamycin (EMD Millipore Corp., Burlington, MA, USA), a specific inhibitor of the mTORC1 complex [27], 30 μM Y-27632(EMD Millipore Corp., Burlington, MA, USA), a ROCK inhibitor [28], or 0.50 μM Z-VAD-FMK, a pan-caspase inhibitor [29].

### 2.4. Generation of Apoptotic Thymocytes

Apoptotic thymocytes were generated by serum starvation as described previously [24]. Around 80% of the resulted cells were Annexin V-positive [30]. Number of cells was determined by Bürker chamber cell counting.

### 2.5. Cell Viability Assay

At day 6, BMDMs were exposed for 24 h to BSA or to various concentrations of BSA-conjugated palmitate. Then, the cells were treated with 10 μL Cell Counting Kit-8 (CCK8) reagents for 1 h at 37 °C in the dark. The absorbance of wells was measured at 450 nm in a BioTek microplate reader (Agilent Technologies, Santa Clara, CA, USA).

### 2.6. Efferocytosis Assays

Percentage of the apoptotic thymocyte engulfing BMDMs exposed to various compounds was determined as it was described previously [24].

### 2.7. Fluorescent Microscopy

Macrophages were stained with 5 µM 5(6)-Carboxyfluorescein diacetate N-succinimidyl ester for 24 h. Apoptotic thymocytes were stained with 2.5 µM Cell CellTracker™ Deep Red Dye (Invitrogen, Carlsbad, CA, USA) for 24 h, and were then added to 2 × 10^5^ C57BL/6 macrophages in 1:5 macrophage:target cell ratio for 40 min, then the remaining cells were washed away. Following phagocytosis, BMDMs were fixed by 1% paraformaldehyde. Pictures were taken on fluorescent microscope (4471136, FLoid™ Cell Imaging Station, ThermoFisher, Waltham, MA, USA).

### 2.8. Quantitative Real-Time Polymerase Chain Reaction (qRT-PCR) Analysis of mRNA Expression

Total RNA was isolated from BMDMs cultured alone or exposed to various compounds for 24 h using the TRI reagent (ThermoFisher, Waltham, MA, USA) according to the manufacturer’s guidelines. Total RNA was reverse-transcribed into cDNA using a High-Capacity cDNA Reverse Transcription Kit (Life Technologies, Budapest, Hungary) according to the manufacturer’s instructions. qRT-PCR was carried out in triplicate using pre-designed FAM-labeled MGB assays (Life Technologies, Budapest, Hungary) including LightCycler 480 Multiwell 384 white plates sealed with adhesive tapes on a Roche LightCycler LC 480 real-time PCR instrument. Relative mRNA levels were calculated using the comparative CT method and were normalized to Glyceraldehyde-3-Phosphate Dehydrogenase (GAPDH) mRNA. Catalogue number of the TaqMan assays used for integrin β3, integrin β5, CD36, complement 1q, MFG-E8, Mer tyrosine kinase, Axl tyrosine kinase transglutaminase 2 (TG2), Thrombospondin-1, Tim4, CD14, stabilin2, ELMO, and GAPDH were Mm00443980_m1, Mm00439825_m1, Mm00432403_m1, Mm00437836_m1, Mm00437221_m1, Mm00500549_m1, Mm00434920_m1, Mm00436979_m1, Mm00449032_g1, Mm00432403_m1, Mm00724709_m1, Mm00454684_m1, Mm00523400_m1, and Mm99999915_g1, respectively.

### 2.9. Western Blot Analysis

To obtain the total cellular proteins, cells were harvested and lysed in cold lysis buffer (50 mM Tris–HCl; 0.5% Triton X-100; 1 mM EDTA; 17 mM 2-mercaptoethanol (pH:8.3) and proteinase inhibitors). The protein content of the samples was determined by Bio-Rad Protein Assay Dye (Bio-Rad, Budapest, Hungary), and then the supernatant was boiled in loading buffer with an aliquot corresponding to 20–40 μg of protein. BMDM lysates were run on SDS polyacrylamide gels, and the separated proteins were electroblotted onto polyvinylidene difluoride membranes. Membranes were probed overnight at 4 °C with monoclonal anti-mouse p70 S6 Kinase (Cell Signaling Technology, Inc., Beverly, MA, USA), P-p70 S6 Kinase (T389) (Cell Signaling Technology, Inc., Beverly, MA, USA), Akt (pan) (Cell Signaling Technology, Inc., Beverly, MA, USA), P-Akt (Ser473) (Cell Signaling Technology, Inc., Beverly, MA, USA), myosin phosphatase-targeting subunit-1 (MYPT1) (ThermoFisher, Waltham, MA, USA), and P-MYPT1 (ThermoFisher, Waltham, MA, USA), Rock1 (MyBioSource, Inc., San Diego, CA, USA, AMPKα (Cell Signaling Technology, Inc., Beverly, MA, USA), P-AMPKα (Thr172) (Cell Signaling Technology, Inc., Beverly, MA, USA) antibodies in 1:1000, and monoclonal anti-β-actin antibodies (A5441) in 1:5000 dilutions. After three washes with TBS-T, the membrane was incubated for 1 h with Anti-Mouse IgG (whole molecule)-Peroxidase antibody produced in sheep or with Goat-a-rabbit IgG(H+L) HRP Conjugate Secondary Antibody in 1:10000 dilutions followed by enhanced chemiluminescence (Advansta Inc., San Jose, CA, USA).

### 2.10. Determination of Rac1, Cdc42 and RhoA Activity in Macrophages

Macrophages from 2 wells cultured under the same conditions were collected as one sample, and 2.8 × 10^6^ from them were used for the determinations. The activity of the three G proteins was then determined by the G-LISA Rac1, Cdc42, and RhoA activation assay kits (Cytoskeleton Inc. Denver, CO, USA) according to the manufacturer’s instructions.

### 2.11. Staining of Engulfing BMDMs for Confocal Lase-Scanning Microscopy

Macrophages on μ-Slide 8 Well ibiTreat plates were stained with 50 nM LysoTracker™ Green DND-26 (Invitrogen, Carlsbad, CA, USA) for 1 h. Apoptotic thymocytes were stained with 2.5 µM Cell CellTracker™ Deep Red Dye (Invitrogen, Carlsbad, CA, USA) for 24 h and were then added to 6 × 10^4^ C57BL/6 macrophages in 1:5 macrophage:target cell ratio for 40 min; then, the remaining cells were washed away. Confocal images were taken without fixing the cells.

### 2.12. Confocal Laser Scanning Microscopy

Fluorescent images were recorded on a Nikon A1 Eclipse Ti2 confocal laser-scanning microscope (Nikon, Tokyo, Japan) using a Plan Apo 60× water objective [NA = 1.27]. Laser lines of 488 nm and 647 nm were used for the excitation of LysoTracker^®^ Green DND-26 and CellTracker™ Deep Red Dye, and the fluorescence emissions were detected through bandpass filters of 500–550 nm and 660–740 nm, respectively. Images of approximately 1 μm-thick optical sections, each containing 512 × 512-pixels, with pixel size 100 or 410 nm, were acquired with 2.2 µs pixel dwell time. Images were taken in sequential mode to minimize crosstalk between the channels, and an average of 4 lines was used for denoising the images.

### 2.13. Statistical Analysis

All the data are representative of at least three independent experiments carried out with BMDMs originated from three different mice and apoptotic cells exposed to them generated also from three different mice. Values are expressed as mean ± S.D. For differences between 2 groups, two-tailed unpaired Student’s t-test was used, and for comparisons *n* > 2 groups one-way ANOVA (with Tukey’s multiple comparisons test) was used. All statistical analyses were performed using GraphPad Prism 6.01, and a *p* value <0.05 was considered as significant and is indicated by asterisk (*).

## 3. Results

### 3.1. Palmitate Exposure Inhibits Phagocytosis of Apoptotic Cells by BMDMs without Significantly Affecting the mRNA Expression of Various Phagocytosis-Related Molecules

To test whether palmitate indeed inhibits efferocytosis by BMDMs, BMDMs were exposed to increasing concentrations of palmitate for 24 h, then their phagocytic capacity was tested by exposing them to apoptotic thymocytes for 40 min.

**Figure 1:**
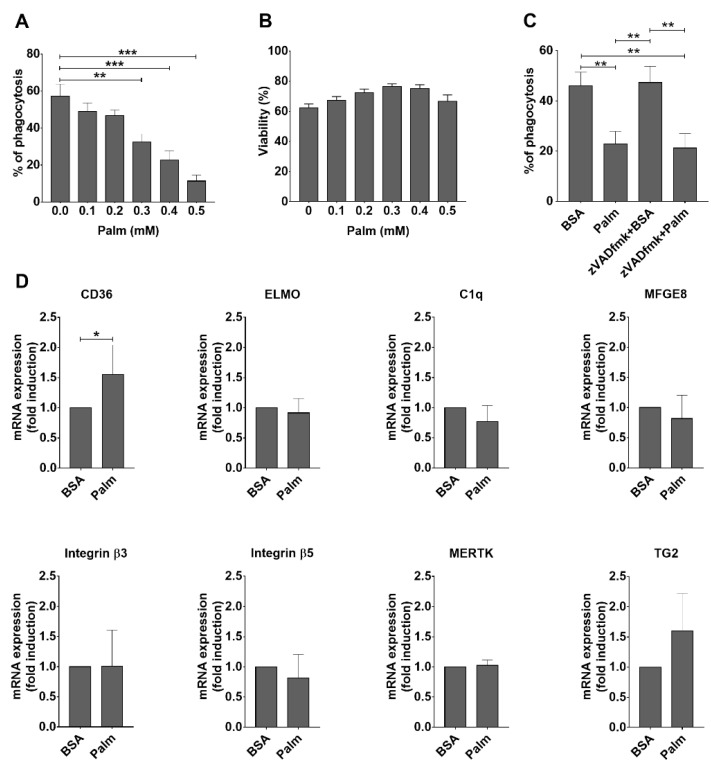
Palmitate inhibits engulfment of apoptotic thymocytes by BMDMs without affecting the mRNA expression of key phagocytic receptors or bridging molecules. (**A**) Palmitate inhibits efferocytosis of BMDMs in a dose-dependent manner. (**B**) Palmitate does not affect the viability of BMDMs up to 0.4 mM concentration tested by the Cell Counting Kit (CCK8). (**C**) Z-VAD-FMK (50 μM), an apoptosis inhibitor, does not prevent palmitate (0.4 mM)-induced inhibition of efferocytosis by BMDMs. (**D**) mRNA expression of various phagocytic receptors and bridging molecules in macrophages exposed or not to 0.4 mM palmitate for 24 h determined by qRT-PCR. GAPDH was used as a reference gene. All data are expressed as mean ± SD (*n* = 3). Asterisks indicate statistically significant difference (* *p* < 0.05, ** *p* < 0.01, and *** *p* < 0.001).

As shown in Figure 1A, palmitate inhibited efferocytosis by BMDMs in a concentration-dependent manner confirming previous observations [20]. Previous studies indicated that palmitate can trigger endoplasmic reticulum (ER) stress, ceramide production, and oxidative stress leading to apoptotic death of macrophages [31,32]. Apoptosis of palmitate-exposed macrophages could explain the observed decreased efferocytosis capacity. However, tested by the Cell Counting Kit (CCK8), palmitate in the concentration range, at which it significantly inhibited efferocytosis, did not affect the viability of macrophages (Figure 1B). Since apoptosis induction is also time-dependent, to make sure that we remain in the concentration range at which palmitate does not trigger apoptosis, we selected 0.4 mM concentration of palmitate exposure for further studies. Indeed, Z-VAD-FMK, a pan-caspase (and consequently apoptosis) inhibitor, did not improve the phagocytic capacity of BMDMs exposed to 0.4 mM palmitate (Figure 1C), proving that the decreased phagocytic capacity of macrophages is not related to a possible palmitate-induced apoptosis at this concentration of the compound. 

Next, we checked whether palmitate affects the mRNA expression of key molecules participating in efferocytosis. However, as partially shown in Figure 1D, the mRNA expressions of the tested efferocytosis-related molecules (integrin β3, integrin β5, transglutaminase 2, thrombospondin-1, MFG-E8, Mer tyrosine kinase, Axl tyrosine kinase, complement 1q, Tim4, CD14, stabilin-2, ELMO) were not altered in BMDMs upon exposure to palmitate, with the exception of that of CD36, which participates also in fatty acid uptake, and the expression of which is known to be regulated by the fatty acid sensor transcription factor peroxisome proliferator-activated receptor (PPAR)γ [33]. Thus, we decided to search for other mechanisms.

### 3.2. Exposure to Palmitate Affects Efferocytosis Very Likely via the Energy Sensing Mechanisms in BMDMs

**Figure 2:**
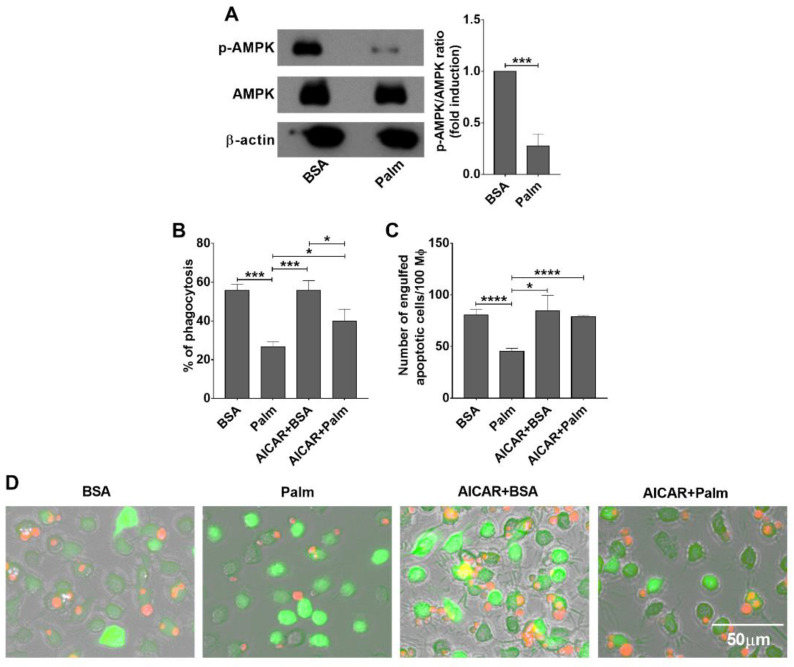
Exposure of BMDMs to palmitate results in a decrease in AMPK activity leading to inhibition of efferocytosis. (**A**) Exposure to 0.4 mM palmitate for 24 h leads to a decreased Thr^172^ phosphorylation of AMPK in BMDMs detected by Western blot analysis. β-actin was used as a loading control. One representative gel of three is shown. AICAR (0.5 mM), an AMPK activator, attenuates the efferocytosis inhibition of BMDMs induced by palmitate (0.4 mM) exposure for 24 h detected by (**B**) determining the percentage of engulfing macrophages by FACS analysis or by (**C**) counting the number of engulfed cells. All data are expressed as mean ± SD (*n* = 4). Asterisks indicate statistically significant difference (* *p* < 0.05, *** *p* < 0.001, and **** *p* < 0.0001). (**D**) Representative fluorescent microscopic images of macrophages (green) engulfing apoptotic thymocytes (red) following 40 min of efferocytosis in the presence or absence of 0.4 mM palmitate with or without AICAR.

Exposure to high amounts of palmitate increases the energy load of macrophages. AMPK is an important sensor and integrator of signals that control energy balance through the regulation of multiple biochemical pathways in all eukaryotes [34]. AMPK is an αβγ heterotrimer, the maximal activation of which requires phosphorylation of threonine 172 (Thr^172^) in the activation loop of the kinase domain [35]. Its activity is increasing when cells lack sufficient energy, while it is inhibited, when cells are exposed to sufficient amount of nutrients. Indeed, Thr^172^ phosphorylation of AMPKα detected by Western blot analysis decreased following administration of palmitate (Figure 2A) indicating its decreasing enzymatic activity. Thus, we checked whether mimicking low energy amount status by activating AMPK could alter palmitate exposure-induced inhibition of efferocytosis by BMDMs. As shown in Figure 2B–D, preincubation of palmitate-exposed macrophages with AICAR, a widely used pharmacological AMPK activator, for 1.5 h prior to efferocytosis significantly enhanced the phagocytic capacity of palmitate exposed BMDMs. These data indicate that long-term exposure of BMDMs to palmitate decreases the activity of AMPK, and preventing the loss of AMPK activity can prevent the palmitate-induced decrease in the efferocytosis activity of macrophages.

### 3.3. Palmitate Exposure Triggers mTORC1 Activity in BMDMs

**Figure 3:**
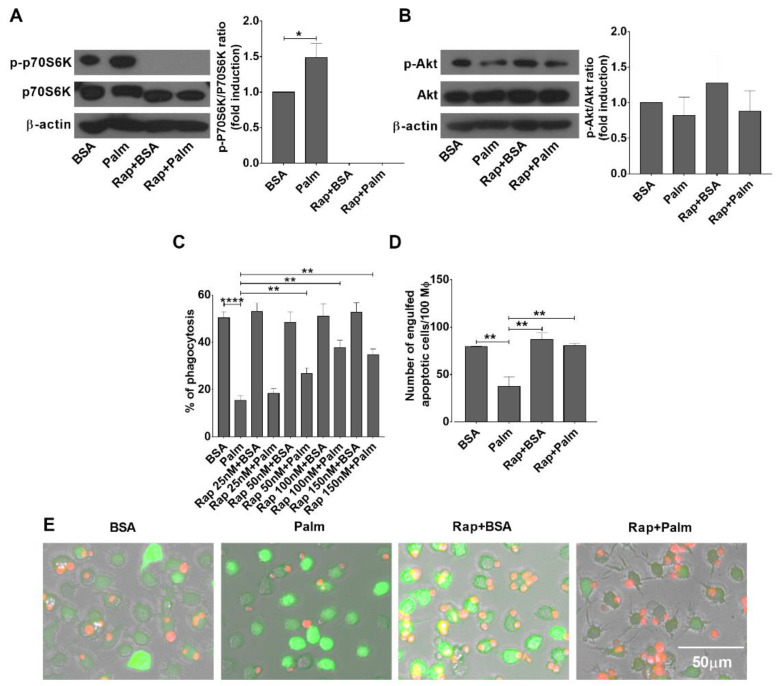
Exposure of BMDMs to palmitate results in mTORC1 activation leading to inhibition of efferocytosis. (**A**) Exposure of BMDMs to 0.4 mM palmitate for 24 h leads to an increased Thr^389^ phosphorylation of p70S6K1, a downstream substrate of mTORC1, while it decreases following exposure to rapamycin (100 nM), detected by Western blot analysis. β-actin was used as a loading control. One representative gel of three is shown. (**B**) Exposure of BMDMs to 0.4 mM palmitate or to rapamycin (100 nM) for 24 h does not affect the Ser^473^ phosphorylation of Akt, a substrate of mTORC2 in BMDMs, detected by Western blot analysis. β-actin was used as a loading control. One representative gel of three is shown. Rapamycin (100 nM), an mTORC1 inhibitor, attenuates the efferocytosis inhibition of BMDMs induced by palmitate exposure for 24 h detected by (**C**) determining the percentage of engulfing macrophages by FACS analysis or by (**D**) counting the number of engulfed cells. All data are expressed as mean ± SD (*n* = 4). Asterisks indicate statistically significant difference (* *p* < 0.05, ** *p* < 0.01, and **** *p* < 0.001). (**E**) Representative fluorescent microscopic images of macrophages (green) engulfing apoptotic thymocytes (red) following 40 min of efferocytosis in the presence or absence of 0.4 mM palmitate with or without 100 nM rapamycin.

Though one way AMPK activation could alter palmitate metabolism in palmitate-exposed macrophages is promoting its mitochondrial oxidation [34], AMPK also interacts with mTOR and regulates mTOR activity to mediate its effects. mTOR, forming two functional complexes (mTORC1 and mTORC2), is a serine/threonine protein kinase which regulates intracellular signaling related to cell growth, cell survival, and protein synthesis [36]. AMPK was reported to inhibit mTORC1 [13], while to activate mTORC2 activities [37]. Thus, we decided to check whether exposure to palmitate alters mTOR activation in macrophages. As shown in Figure 3A, exposure of BMDMs to palmitate triggered mTORC1 activation detected by the Thr^389^ phosphorylation of the 70 kDa ribosomal protein S6 kinase 1 (p70S6K1), a downstream substrate of mTORC1 [38], while it did not significantly affect the activity of mTORC2 detected by the Ser^473^ phosphorylation of Akt [39] (Figure 3B).

To decide whether mTORC1 activation participates in the regulation of palmitate-induced inhibition of efferocytosis, we tested the effect of increasing concentrations of rapamycin, a selective inhibitor of mTORC1, on the efferocytosis capacity of palmitate-exposed macrophages. As seen in Figure 3C, rapamycin prevented palmitate-induced inhibition of efferocytosis in BMDMs in a concentration-dependent manner. At its most effective 100 nM concentration, administration of rapamycin resulted in full inhibition of p70S6K1 phosphorylation, while it had no effect on the phosphorylation of Akt (Figure 3A,B). These data indicate that exposure to palmitate induces mTORC1 activation in BMDMs, and administration of rapamycin indeed selectively inhibits its activity. What is more, as shown in Figure 3D,E, inhibition of mTORC1 activity in the presence of palmitate results in the same number of apoptotic cell uptake as in the absence of it.

### 3.4. Palmitate Exposure Triggers an mTORC1 Dependent Rho Kinase Activation to Inhibit Efferocytosis

**Figure 4:**
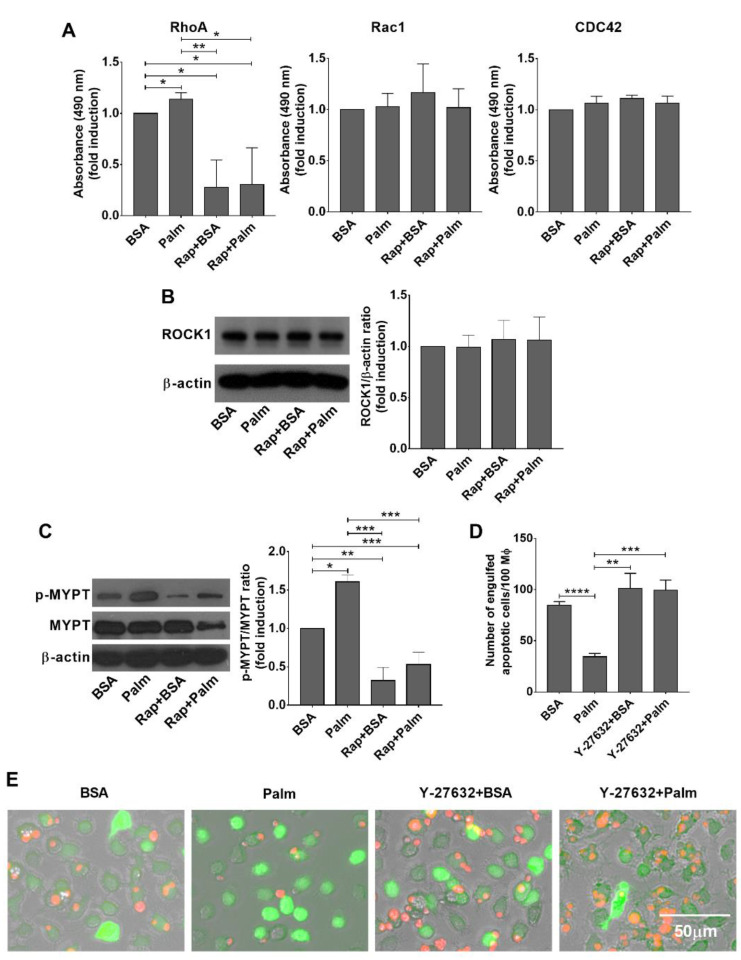
Exposure of BMDMs to palmitate results in an mTORC1-dependent ROCK1 activation leading to inhibition of efferocytosis. (**A**) Exposure of BMDMs to 0.4 mM palmitate for 24 h leads to enhanced mTORC1-dependent RhoA activity detected by G-LISA RhoA activation assay kit, while the activity of Rac1 and Cdc42 did not alter. (**B**) Exposure of BMDMs to 0.4 mM palmitate for 24 h did not affect the ROCK1 protein expression levels detected by Western blot analysis. β-actin was used as a loading control. One representative gel of three is shown. (**C**) Exposure to 0.4 mM palmitate for 24 h leads to an increased mTORC1-dependent Thr^850^ phosphorylation of MYPT-1, a downstream substrate of ROCK1 detected by Western blot analysis, while addition of rapamycin (100 nM) reverses this effect. β-actin was used as a loading control. One representative gel of three is shown. (**D**) Y-27632 (30 μM), a ROCK inhibitor, reverses the efferocytosis inhibition of BMDMs induced by palmitate exposure for 24 h detected by counting the number of engulfed cells. All data are expressed as mean ± SD (*n* = 4). Asterisks indicate statistically significant difference (* *p* < 0.05, ** *p* < 0.01, *** *p* < 0.001, **** *p* < 0.001). (**E**) Representative fluorescent microscopic images of macrophages (green) engulfing apoptotic thymocytes (red) following 40 min of efferocytosis in the presence and absence of 0.4 mM palmitate with or without 30 μM Y-27632.

As exposure of palmitate did not affect the expression of phagocytic receptors (Figure 1D), it was very likely that palmitate-induced mTORC1 signaling interfered with the efferocytosis signaling pathways. Since G proteins play a crucial role in the regulation of cytoskeletal rearrangements during efferocytosis [5], we tested the activity of Rac1, Cdc42, and RhoA in the BMDMs by G-LISA Rac1, Cdc42, and RhoA activation assay kits following 24 h exposure to palmitate. As shown in Figure 4A, while exposure to palmitate did not affect the activity of Rac1 or Cdc42, it significantly enhanced that of RhoA. In addition, exposure of BMDMs to rapamycin did not affect Rac1 or Cdc42 activity alone or in the presence of palmitate, but decreased the activity of RhoA indicating an mTORC1-dependent regulation of RhoA activity.

ROCK is an effector of the GTPase Rho, and belongs to the AGC family of kinases [40]. Since previous studies indicated a negative role of RhoA/ROCK1 signaling in the efferocytosis [6], we checked the protein level and the activity of ROCK1 in palmitate exposed BMDMs. As seen in Figure 4B, exposure to palmitate, rapamycin, or palmitate and rapamycin did not affect the protein levels of ROCK1 detected by Western blot analysis, despite of the fact rapamycin was reported to interfere with the translation of ROCK1 at least in mouse peritoneal macrophages [41].

ROCK1 phosphorylates several substrates that play determinant roles in cytoskeletal rearrangements, including myosin phosphatase-targeting subunit-1 (MYPT-1) [42]. In agreement with the RhoA activation results (Figure 4A), Western blot analysis of lysates of BMDMs showed that Thr^850^ phosphorylation of MYPT-1 was significantly enhanced in palmitate-exposed BMDMs, and rapamycin treatment significantly decreased the level of this phosphorylation (Figure 4C). Altogether, these data indicate that mTORC1 is capable of activating RhoA and consequently ROCK1, and palmitate might enhance ROCK1 activity in an mTORC1-dependent manner.

If activation of ROCK1 plays a determinant role in the palmitate-induced inhibition of efferocytosis by BMDMs, one would expect a reversal of this inhibition by inhibiting ROCK1. Indeed, as shown in Figure 4D and E, Y-27632, a specific inhibitor of ROCK activity, could also reverse the inhibition of palmitate induced efferocytosis. However, we could not confirm the previously published enhancing effect of ROCK inhibition on the basal efferocytosis capacity of macrophages [6], at least in BMDMs.

### 3.5. Palmitate Exposure Induces Autophagy but Inhibits the Fusion of Autophagic Vacuoles and Lysosomes

Previous studies have demonstrated that exposure of various cells to palmitate induces endoplasmic reticulum stress and, consequently, an unfolded protein response (UPR) in them [43]. There are three UPR sensors with distinct activation mechanisms, which trigger different signaling pathways but also activate common adaptive processes for cell survival including autophagy [44,45,46].

On a molecular level, several functional complexes are involved in autophagosome biogenesis, cargo recognition, and in mediating autophagosome–lysosome fusion. Among them, an ubiquitin-like system is required for specific cargo recognition, namely the ATG8-conjugation system. It serves to lipidate ATG8 family members, including microtubule-associated protein 1 light chain 3 (MAP1LC3 or LC3) [47,48]. The lipidated form of LC3 (LC3-II) is membrane-bound, and its increased amount is used to demonstrate enhanced autophagy [49,50]. During selective autophagy, various autophagy receptors mediate selectivity by being able to interact with the cargo on one hand and with the autophagy machinery on the other hand. One of them is p62, which specifically links the cargo to the membrane-bound LC3 [51]. During the autophagy process, p62 itself is constantly degraded together with the selected cargo; therefore, decreased levels of p62 also indicate activation of the autophagy pathway [52].

**Figure 5:**
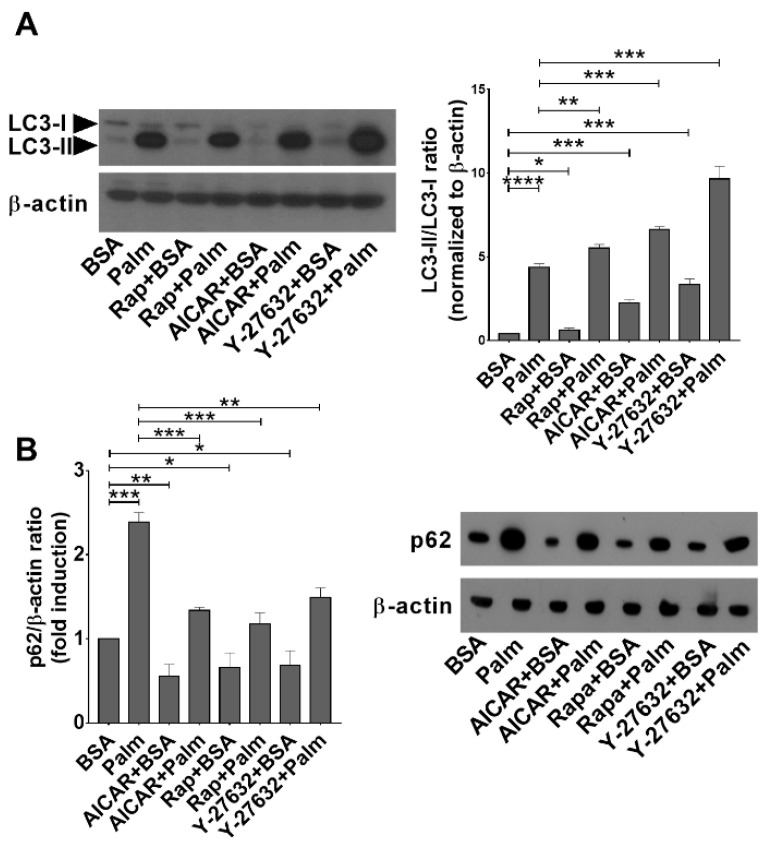
Palmitate exposure induces autophagy but inhibits the fusion of autophagosomes and lysosomes in BMDMs. Administration of AICAR, rapamycin, or Y-27632 interferes with this inhibition. (**A**) Expression of LC3I and LC3II in BMDMs exposed to palmitate for 24 h alone or together with rapamycin (100 nM), AICAR (0.5 mM), or Y-27632 (30 μM) determined by Western blot analysis. β-actin was used as a loading control. One representative gel of three is shown. (**B**) Expression of p62 in BMDMs exposed to palmitate alone or together with rapamycin (100 nM), AICAR (0.5 mM), or Y-27632 (30 μM) determined by Western blot analysis. β-actin was used as a loading control. One representative gel of three is shown. All data are expressed as mean ± SD (*n* = 3). Asterisks indicate statistically significant difference (* *p* < 0.05, ** *p* < 0.01, *** *p* < 0.001, **** *p* < 0.0001).

Since the mTORC1 pathway is a known inhibitor of autophagy [13], while palmitate is known to induce autophagy via an mTORC1-independent pathway [43], we decided to investigate the autophagic process in palmitate-exposed BMDMs. As shown in Figure 5A, exposure of BMDMs to rapamycin, AICAR, or Y-27632 alone increased the ratio of LC3-II/LC3-I protein amounts, indicating that these compounds acted as autophagy inducers in BMDMs. In accordance, the amount of p62 decreased in the presence of these compounds (Figure 5B). Exposure of BMDMs to palmitate for 24 h significantly enhanced the amount of LC3-II in macrophages, indicating an enhanced autophagy response to palmitate as well (Figure 5A). However, in contrast to the three compounds, palmitate exposure increased the level of p62 (Figure 5B) in BMDMs indicating a delay or block in the autophagosome–lysosome fusion leading to the accumulation of LC3II positive autophagosomes. Exposure of palmitate-treated BMDMs to rapamycin, AICAR, or Y-27632 significantly reduced the palmitate-induced p62 levels, indicating that these compounds reversed the palmitate-induced blockade of autophagosome–lysosome fusion (Figure 5B).

**Figure 6:**
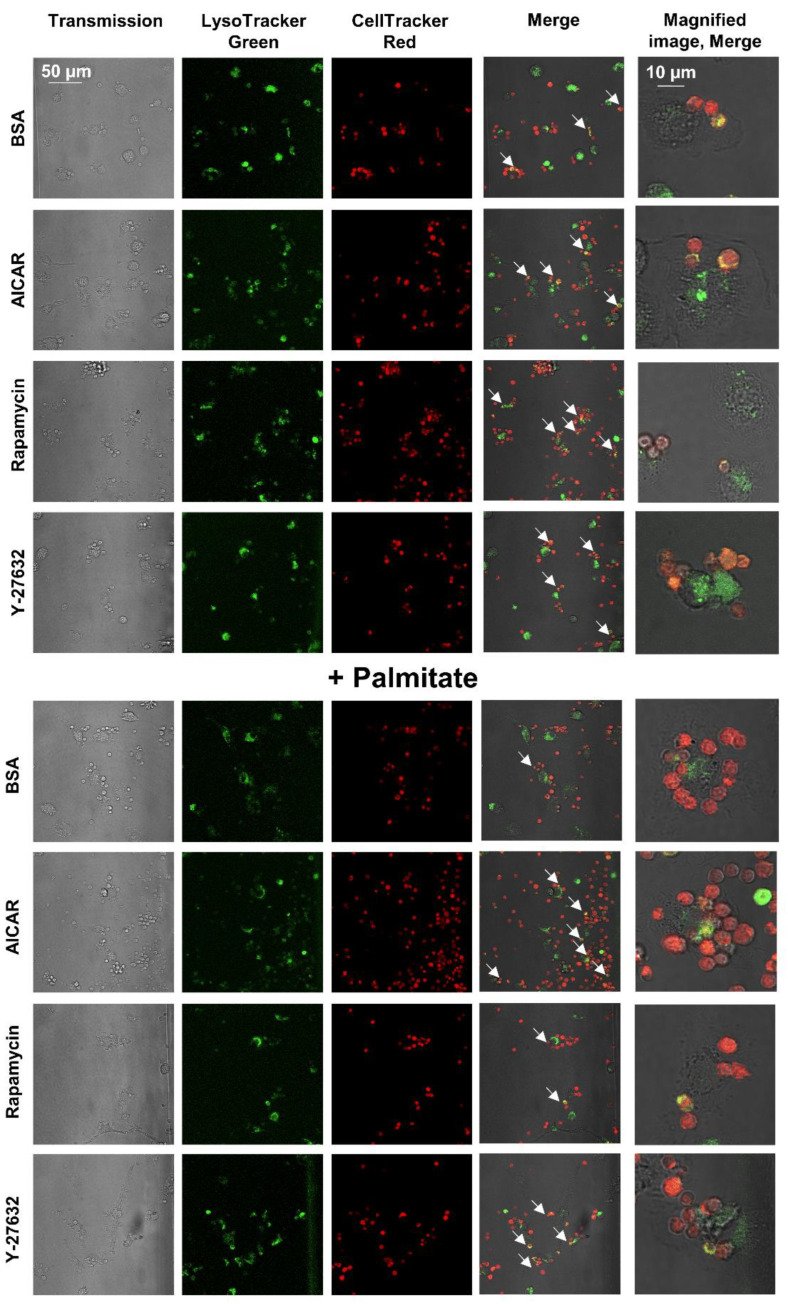
Delayed appearance of apoptotic cells in the phagolysosomes of BMDMs triggered by 24-h palmitate exposure is reverted by administration of AICAR, rapamycin, or Y-27632. Appearance of Deep Red Dye-stained red apoptotic thymocytes in the LysoTracker^TM^ Green-stained green phagolysosomes of BMDMs exposed to palmitate (0.4 mM) for 24 h alone or together with rapamycin (100 nM), AICAR (0.5 mM), or Y-27632 (30 μM) detected with confocal microscopy. Yellow/orange colors (arrows) indicate apoptotic cells appearing in the phagolysosomes following fusion of phagosomes with lysosomes.

Interestingly, when uptake of apoptotic cells (Figure 6) was followed by LysoTracker green-stained BMDMs, we could hardly detect apoptotic cells within the phagolysosomes of palmitate-exposed BMDMs. While out of 100 macrophages containing apoptotic cells, we detected yellow color in 92 cases; this was only 24 in the presence of palmitate. This observation indicates that prolonged ROCK signaling might interfere with the phagosome–lysosome fusion as well. However, we cannot exclude the alternative possibility that only the delayed phagocytic uptake of apoptotic cells explains their delayed appearance in the phagolysosomes.

## 4. Discussion

Increasing evidence indicates that if the clearance of apoptotic cells is improper, it can act as a contributor to the development of various chronic inflammatory diseases [2]. This is related to the fact on one side that the accumulating uncleared apoptotic cells undergo secondary necrosis and trigger inflammation, and on the other side, while properly engulfing macrophages activate anti-inflammatory mechanisms that prevent tissue inflammation, these mechanisms are broken if efferocytosis is impaired.

Obesity is characterized by low-grade inflammation that originates from the expanding adipose tissue and the enhanced death of lipid-loaded adipocytes, which trigger inflammation in the recruited adipose tissue macrophages due to the enhanced adipocyte lipid release [52,53]. Obesity is known to predispose to a variety of co-morbidities and complications characterized by chronic inflammation that affect overall health. Thus, obese individuals are prone to develop atherosclerosis [21], chronic obstructive pulmonary disease [22], various chronic rheumatic diseases [23], or oral inflammations [54]. Though there is a general belief that the sensitivity to develop other chronic inflammatory diseases is related to the increased pro-inflammatory cytokine or adipokine levels associated with obesity [52,53], obesity is also characterized with impaired clearance of apoptotic cells, due to high levels of circulating saturated fatty acids, such as palmitate, which interfere with the efferocytosis process [20]. Thus, the resulting impaired phagocytosis of apoptotic cells everywhere within the body might also contribute to the increased ability of obese individuals to develop chronic inflammatory diseases.

Macrophages and efferocytosis by macrophages also play a central role in guiding skeletal muscle regeneration following injury [55,56] or wound healing [57]. As a result, improper efferocytosis by palmitate-exposed macrophages might also contribute to the improper skeletal muscle regeneration or wound healing observed in obese individuals [58,59]. Consequently, understanding how saturated fatty acids affect efferocytosis, and reversing the efferocytosis inhibition might have beneficial effects in the treatment of obese patients.

In the present study, we confirmed that elevated levels of palmitate interfere with the clearance of apoptotic cells by BMDMs. Palmitate exposure did not affect the expression of key phagocytic receptors, but it led to a defective autophagy resulting in accumulation of the cell membranes in the autophagosomes and, consequently, in an inadequate supply of membranes for the efferocytosis process. The defective autophagy was the result of the activation of an mTORC1 signaling pathway that inhibited the fusion of lysosomes with the autophagosomes produced via the endoplasmic reticulum stress-triggered, mTORC1-independent autophagy process [43]. According to our results, the enhanced mTORC1 activation in palmitate-exposed BMDMs is very likely the result of the inhibition of AMPK activity either due to low levels of AMP in the presence of the high energy-containing palmitate or, as we demonstrated, due to the decreased Thr^172^ phosphorylation of the enzyme. In accordance with our findings, palmitate was shown to activate protein phosphatase 2A activity leading to AMPK inhibition in endothelial cells [60], and palmitate was shown to activate the mTORC1 pathway also in myoblasts in an AMPK-dependent manner [61]. In addition, palmitate exposure was reported to enhance mTORC1-dependent p70S6K1 activation in hepatocytes [62] and podocytes [63] as well. The mechanisms through which inhibition of AMPK activity led to mTORC1 activation in palmitate-exposed BMDMs was not investigated in our studies, but AMPK is known to inhibit mTORC1 activity via phosphorylation of both tuberous sclerosis complex (TSC) 2 [64] and Raptor [65]. Involvement of an AMPK-regulated mTORC1 pathway in the palmitate-induced inhibition of efferocytosis was demonstrated by the reversal of this inhibition by AICAR, an activator of AMPK, and rapamycin, a selective inhibitor of mTORC1.

While the expressions of efferocytosis-related molecules were not altered by administration of palmitate, palmitate exposure increased the activity of RhoA, one of the key G proteins regulating efferocytosis in BMDMs, and the mTORC1 inhibitor rapamycin inhibited it. The potential involvement of the RhoA pathway in the palmitate-induced inhibition of efferocytosis was further demonstrated by the increased ROCK1 activity following palmitate exposure, by its decreased activity in the presence of rapamycin, and by the reversal of the palmitate-induced inhibition of efferocytosis by Y-27632, a specific ROCK inhibitor. Our finding that mTORC1 could be involved in the regulation of the RhoA/Rho kinase signaling pathway is relatively novel, as we found only one publication which ended up with similar findings [66]. In support of our finding, activation of ROCK1 was reported to be linked to the altered lipid metabolism induced by a high fat diet also in hepatocytes [67].

The AMPK/mTORC1 pathway is a known regulator of autophagy, and some elements of autophagy and the noncanonical pathway of efferocytosis overlap [11,12]. However, the mTORC1 pathway does not affect LC3-mediated phagocytosis [12]. That is why we looked at the autophagy process in palmitate-exposed BMDMs. Analysis of autophagy in palmitate-exposed macrophages indicated that despite the fact that palmitate activated the AMPK/mTORC1 pathway, which inhibits several steps of autophagy including the fusion of autophagosomes with the lysosomes [68,69], in accordance with previous publications [43], simultaneously it also triggered an mTORC1-independent autophagy pathway, and the two oppositely acting pathways lead to accumulation of autophagosomes. Addition of AICAR, rapamycin, or Y-27632 to palmitate-exposed macrophages all overcame the inhibition of the fusion detected by the decreasing levels of p62. Our data cannot decide whether AICAR or rapamycin affects the fusion process indirectly via regulating the activity of ROCK, or by affecting AMPK or mTORC1 activities directly, as AMPK [68], mTORC1 [69], and ROCK [70,71] were all reported to affect the autophagosome–lysosome fusion process.

**Figure 7:**
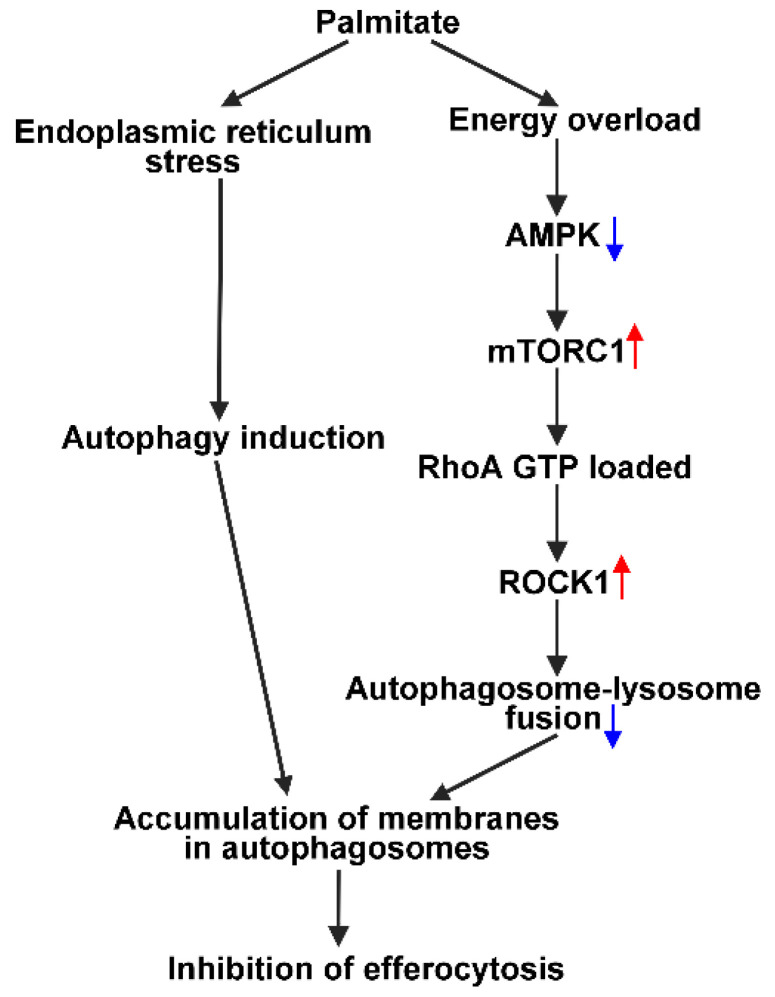
Schematic diagram of signaling pathways induced by palmitate in BMDMs that result in defective autophagy, and consequently in impaired efferocytosis.

The data presented indicate that palmitate induces a defective autophagic flux in BMDMs in which increased production of autophagosomes is not coupled to an enhanced lysosomal degradation. The amount of LC3-II was reported to reflect the number of autophagosomes within the cells [49]; consequently, accumulation of LC3-II in palmitate-exposed BMDMs reflects a large amount of membranes accumulating in the autophagosomes. Phagocytosis, however, must be accompanied by fusion of vesicles at the plasma membrane site of apoptotic cell uptake, which allows growing of the nascent phagosome and serves to compensate for the loss of plasma membrane surface area [72], and the vesicle sources are the endosomes and lysosomes [15,16]. Thus, we propose that inhibition of efferocytosis by palmitate might be the result of an inadequate supply of membranes for the phagocytosis due to the initiation of the defective autophagy (Figure 7). Further studies are required to determine whether ROCK activation also interferes with the phagosome–lysosome fusion process.

Interestingly, activation of AMPK could reverse the inhibition of efferocytosis induced by palmitate, and activators of AMPK are already in use in the treatment of obese patients [73,74,75]. Our data indicate that in addition to the well-known effects of AMPK activation on the metabolism, AMPK activators might also correct the impaired efferocytosis in obese patients. Based on our results, inhibitors of ROCK could also be considered.

Though our studies focused only on BMDMs, our findings on the effects of palmitate in BMDMs might be applicable for other tissues as well. For example, in hepatocytes, high levels of palmitate were reported to activate mTORC1 [62] and ROCK1 [67], and also to induce a defective autophagy with p62 accumulation observed here [76]. Thus, our data presented in our paper indicate that AMPK activators or ROCK inhibitors might have beneficial effects in the treatment of lipotoxicity also in other tissues of obese individuals by influencing the defective autophagy induced by the accumulation of saturated fatty acids.

## Data Availability

Not applicable.

## References

[1] Hart S.P., Dransfield I., Rossi A.G. (2008). Phagocytosis of apoptotic cells. Methods.

[2] Poon I.K., Lucas C.D., Rossi A.G., Ravichandran K.S. (2014). Apoptotic cell clearance: Basic biology and therapeutic potential. Nat. Rev. Immunol..

[3] Barth N.D., Marwick J.A., Vendrell M., Rossi A.G., Dransfield I. (2017). Phagocytic synapse” and clearance of apoptotic cells. Front. Immunol..

[4] Tóth B., Garabuczi E., Sarang Z., Vereb G., Vámosi G., Aeschlimann D., Blaskó B., Bécsi B., Erdődi F., Lacy-Hulbert A. (2009). Transglutaminase 2 is needed for the formation of an efficient phagocyte portal in macrophages engulfing apoptotic cells. J. Immunol..

[5] Nakaya M., Kitano M., Matsuda M., Nagata S. (2008). Spatiotemporal activation of Rac1 for engulfment of apoptotic cells. Proc. Natl. Acad. Sci. USA.

[6] Tosello-Trampont A.C., Nakada-Tsukui K., Ravichandran K.S. (2003). Engulfment of apoptotic cells is negatively regulated by Rho-mediated signaling. J. Biol. Chem..

[7] Kobayashi S., Shirai T., Kiyokawa E., Mochizuki N., Matsuda M., Fukui Y. (2001). Membrane recruitment of DOCK180 by binding to PtdIns(3,4,5)P3. Biochem. J..

[8] Cote J.F., Motoyama A.B., Bush J.A., Vuori K. (2005). A novel and evolutionarily conserved PtdIns(3,4,5)P3-binding domain is necessary for DOCK180 signalling. Nat. Cell Biol..

[9] Croisé P., Estay-Ahumada C., Gasman S., Ory S. (2014). Rho GTPases, phosphoinositides, and actin: A tripartite framework for efficient vesicular trafficking. Small GTPases.

[10] Thi E.P., Reiner N.E. (2012). Phosphatidylinositol 3-kinases and their roles in phagosome maturation. J. Leukoc. Biol..

[11] Teplova I., Lozy F., Price S., Singh S., Barnard N., Cardiff R.D., Birge R., Karantza V. (2013). ATG proteins mediate efferocytosis and suppress inflammation in mammary involution. Autophagy.

[12] Heckmann B.L., Boada-Romero E., Cunha L.D., Magne J., Green D.R. (2017). LC3-associated phagocytosis and inflammation. J. Mol. Biol..

[13] Dossou A.S., Basu A. (2019). The emerging roles of mTORC1 in macromanaging autophagy. Cancers.

[14] Mellman I.S., Plutner H., Steinman R.M., Unkeless J.C., Cohn Z.A. (1983). Internalization and degradation of macrophage Fc receptors during receptor-mediated phagocytosis. J. Cell Biol..

[15] Hackam D.J., Rotstein O.D., Sjolin C., Schreiber A.D., Trimble W.S., Grinstein S. (1998). v-SNARE-dependent secretion is required for phagocytosis. Proc. Natl. Acad. Sci. USA.

[16] Czibener C., Sherer N.M., Becker S.M., Pypaert M., Hui E., Chapman E.R., Mothes W., Andrews N.W. (2006). Ca^2+^ and synaptotagmin VII-dependent delivery of lysosomal membrane to nascent phagosomes. J. Cell Biol..

[17] Yin C., Argintaru D., Heit B. (2019). Rab17 mediates intermixing of phagocytosed apoptotic cells with recycling endosomes. Small GTPases.

[18] Haobam B., Nozawa T., Minowa-Nozawa A., Tanaka M., Oda S., Watanabe T., Aikawa C., Maruyama F., Nakagawa I. (2014). Rab17-mediated recycling endosomes contribute to autophagosome formation in response to Group A *Streptococcus* invasion. Cell Microbiol..

[19] Ravikumar B., Moreau K., Jahreiss L., Puri C., Rubinsztein D.C. (2010). Plasma membrane contributes to the formation of pre-autophagosomal structures. Nat. Cell Biol..

[20] Li S., Sun Y., Liang C.P., Thorp E.B., Han S., Jehle A.W., Saraswathi V., Pridgen B., Kanter J.E., Li R. (2009). Defective phagocytosis of apoptotic cells by macrophages in atherosclerotic lesions of ob/ob mice and reversal by a fish oil diet. Circ. Res..

[21] Rocha V., Libby P. (2009). Obesity, inflammation, and atherosclerosis. Nat. Rev. Cardiol..

[22] Park J.H., Lee J.K., Heo E.Y., Kim D.K., Chung H.S. (2017). The effect of obesity on patients with mild chronic obstructive pulmonary disease: Results from KNHANES 2010 to 2012. Int. J. Chron. Obstruct. Pulmon. Dis..

[23] Gremese E., Tolusso B., Gigante M.R., Ferraccioli G. (2014). Obesity as a risk and severity factor in rheumatic diseases (autoimmune chronic inflammatory diseases). Front. Immunol..

[24] Fige E., Sarang Z., Sós L., Szondy Z. (2022). Retinoids promote mouse bone marrow-derived macrophage differentiation and efferocytosis via upregulating bone morphogenetic protein-2 and Smad3. Cells.

[25] http://www.wklab.org/wp-content/uploads/2016/02/Palmitate-BSA_Prep_SOP_v080624.pdf.

[26] Corton J.M., Gillespie J.G., Hawley S.A., Hardie D.G. (1995). 5-aminoimidazole-4-carboxamide ribonucleoside. A specific method for activating AMP-activated protein kinase in intact cells?. Eur. J. Biochem..

[27] Li J., Kim S.G., Blenis J. (2014). Rapamycin: One drug, many effects. Cell Metab..

[28] Liao J.K., Seto M., Noma K. (2007). Rho kinase (ROCK) inhibitors. J. Cardiovasc. Pharmacol..

[29] Slee E.A., Zhu H., Chow S.C., MacFarlane M., Nicholson D.W., Cohen G.M. (1996). Benzyloxycarbonyl-Val-Ala-Asp (OMe) fluoromethylketone (Z-VAD-FMK) inhibits apoptosis by blocking the processing of CPP32. Biochem. J..

[30] Köröskényi K., Duró E., Pallai A., Sarang Z., Kloor D., Ucker D.S., Beceiro S., Castrillo A., Chawla A., Ledent C.A. (2011). Involvement of adenosine A2A receptors in engulfment-dependent apoptotic cell suppression of inflammation. J. Immunol..

[31] Schilling J.D., Machkovech H.M., He L., Diwan A., Schaffer J.E. (2013). TLR4 Activation under lipotoxic conditions leads to synergistic macrophage cell death through a TRIF-dependent pathway. J. Immunol..

[32] Brookheart R.T., Michel C.I., Schaffer J.E. (2009). As a matter of fat. Cell Metab..

[33] Chen Y., Zhang J., Cui W., Silverstein R.L. (2022). CD36, a signaling receptor and fatty acid transporter that regulates immune cell metabolism and fate. J. Exp. Med..

[34] Steinberg G.R., Kemp B.E. (2009). AMPK in health and disease. Physiol. Rev..

[35] Hawley S.A., Davison M., Woods A., Davies S.P., Beri R.K., Carling D., Hardie D.G. (1996). Characterization of the AMP-activated protein kinase kinase from rat liver and identification of threonine 172 as the major site at which it phosphorylates AMP-activated protein kinase. J. Biol. Chem..

[36] Laplante M., Sabatini D.M. (2012). mTOR signaling in growth control and disease. Cell.

[37] Kazyken D., Magnuson B., Bodur C., Acosta-Jaquez H.A., Zhang D., Tong X., Barnes T.M., Steinl G.K., Patterson N.E., Altheim C.H. (2019). AMPK directly activates mTORC2 to promote cell survival during acute energetic stress. Sci. Signal.

[38] Hornberger T.A., Sukhija K.B., Wang X.R., Chien S. (2007). mTOR is the Rapamycin-Sensitive Kinase that Confers Mechanically-Induced Phosphorylation of the Hydrophobic Motif Site Thr(389) in p70^S6k^. FEBS Lett..

[39] Bozulic L., Hemmings B.A. (2009). PIKKing on PKB: Regulation of PKB activity by phosphorylation. Curr. Opin. Cell Biol..

[40] Amano M., Nakayama M., Kaibuchi K. (2010). Rho-kinase/ROCK: A key regulator of the cytoskeleton and cell polarity. Cytoskeleton.

[41] Fox R., Nhan T.Q., Law G.L., Morris D.R., Liles W.C., Schwartz S.M. (2007). PSGL-1 and mTOR regulate translation of ROCK-1 and physiological functions of macrophages. EMBO J..

[42] Somlyo A.P., Somlyo A.V. (2003). Ca^2+^ sensitivity of smooth muscle and nonmuscle myosin II: Modulated by G proteins, kinases, and myosin phosphatase. Physiol. Rev..

[43] Woodworth-Hobbs M.E., Perry B.D., Rahnert J.A., Hudson M.B., Zheng B., Russ Price S. (2017). Docosahexaenoic acid counteracts palmitate-induced endoplasmic reticulum stress in C2C12 myotubes: Impact on muscle atrophy. Physiol. Rep..

[44] Kouroku Y., Fujita E., Tanida I., Ueno T., Isoai A., Kumagai H., Ogawa S., Kaufman R.J., Kominami E., Momoi T. (2007). ER stress (PERK/eIF2alpha phosphorylation) mediates the polyglutamine-induced LC3 conversion, an essential step for autophagy formation. Cell Death Differ..

[45] Sharma M., Bhattacharyya S., Sharma K.B., Chauhan S., Asthana S., Abdin M.Z., Vrati S., Kalia M. (2017). Japanese encephalitis virus activates autophagy through XBP1 and ATF6 ER stress sensors in neuronal cells. J. Gen. Virol..

[46] Liu C., Yan D.-Y., Wang C., Ma Z., Deng Y., Liu W., Xu B. (2020). IRE1 signaling pathway mediates protective autophagic response against manganese-induced neuronal apoptosis in vivo and in vitro. Sci. Total Environ..

[47] Nakatogawa H., Suzuki K., Kamada Y., Ohsumi Y. (2009). Dynamics and diversity in autophagy mechanisms: Lessons from yeast. Nat. Rev. Mol. Cell Biol..

[48] Shpilka T., Weidberg H., Pietrokovski S., Elazar Z. (2011). Atg8: An autophagy-related ubiquitin-like protein family. Genome Biol..

[49] Kabeya Y., Mizushima N., Ueno T., Yamamoto A., Kirisako T., Noda T., Kominami E., Ohsumi Y., Yoshimori T. (2000). LC3, a mammalian homologue of yeast Apg8p, is localized in autophagosome membranes after processing. EMBO J..

[50] Klionsky D.J., Abdel-Aziz A.K., Abdelfatah S., Abdellatif M., Abdoli A., Abel S., Abeliovich H., Abildgaard M.H., Abudu Y.P., Acevedo-Arozena A. (2021). Guidelines for the use and interpretation of assays for monitoring autophagy (4th edition). Autophagy.

[51] Rogov V., Dotsch V., Johansen T., Kirkin V. (2014). Interactions between autophagy receptors and ubiquitin-like proteins form the molecular basis for selective autophagy. Mol. Cell.

[52] Alkhouri N., Gornicka A., Berk M.P., Thapaliya S., Dixon L.J., Kashyap S., Schauer P.R., Feldstein A.E. (2010). Adipocyte apoptosis, a link between obesity, insulin resistance, and hepatic steatosis. J. Biol. Chem..

[53] Hotamisligil G.S. (2006). Inflammation and metabolic disorders. Nature.

[54] Pischon N., Heng N., Bernimoulin J.P., Kleber B.M., Willich S.N., Pischon T. (2007). Obesity, inflammation, and periodontal disease. J. Dent. Res..

[55] Chazaud B. (2020). Inflammation and skeletal muscle regeneration: Leave it to the macrophages. Trends Immunol..

[56] Al-Zaeed N., Budai Z., Szondy Z., Sarang Z. (2021). TAM kinase signaling is indispensable for the proper skeletal muscle regeneration process. Cell Death Dis..

[57] Minutti C.M., Knipper J.A., Allen J.E., Zaiss D.M. (2017). Tissue-specific contribution of macrophages to wound healing. Semin. Cell Dev. Biol..

[58] Akhmedov D., Berdeaux R. (2013). The effects of obesity on the skeletal muscle regeneration. Front. Physiol..

[59] Pierpont Y.N., Dinh T.P., Salas R.E., Johnson E.L., Wright T.G., Robson M.C., Payne W.G. (2014). Obesity and surgical wound healing: A current review. ISRN Obes..

[60] Wu Y., Song P., Xu J., Zhang M., Zou M.H. (2007). Activation of protein phosphatase 2A by palmitate inhibits AMP-activated protein kinase. J. Biol. Chem..

[61] Kwon B., Querfurth H.W. (2015). Palmitate activates mTOR/p70S6K through AMPK inhibition and hypophosphorylation of raptor in skeletal muscle cells: Reversal by oleate is similar to metformin. Biochimie.

[62] Mordier S., Iynedjian P.B. (2007). Activation of mammalian target of rapamycin complex 1 and insulin resistance induced by palmitate in hepatocytes. Biochem. Biophys. Res. Commun..

[63] Yasuda M., Tanaka Y., Kume S., Morita Y., Chin-Kanasaki M., Araki H., Isshiki K., Araki S., Koya D., Haneda M. (2014). Fatty acids are novel nutrient factors to regulate mTORC1 lysosomal localization and apoptosis in podocytes. Biochim. Biophys. Acta.

[64] Inoki K., Zhu T., Guan K.L. (2003). TSC2 mediates cellular energy response to control cell growth and survival. Cell.

[65] Gwinn D.M., Shackelford D.B., Egan D.F., Mihaylova M.M., Mery A., Vasquez D.S., Turk B.E., Shaw R.J. (2008). AMPK phosphorylation of raptor mediates a metabolic checkpoint. Mol. Cell.

[66] Peterson T.R., Laplante M., van Veen E., van Vugt M., Thoreen C.C., Sabatini D.M. (2015). mTORC1 regulates cytokinesis through activation of Rho-ROCK signaling. arXiv.

[67] Huang H., Lee S.H., Sousa-Lima I., Kim S.S., Hwang W.M., Dagon Y., Yang W.M., Cho S., Kang M.C., Seo J.A. (2018). Rho-kinase/AMPK axis regulates hepatic lipogenesis during overnutrition. J. Clin. Investig..

[68] Jang M., Park R., Kim H., Namkoong S., Jo D., Huh Y.H., Jang I.S., Lee J.I., Park J. (2018). AMPK contributes to autophagosome maturation and lysosomal fusion. Sci. Rep..

[69] Huang H., Ouyang Q., Zhu M., Yu H., Mei K., Liu R. (2021). mTOR-mediated phosphorylation of VAMP8 and SCFD1 regulates autophagosome maturation. Nat. Commun..

[70] Miyazaki M., Hiramoto M., Takano N., Kokuba H., Takemura J., Tokuhisa M., Hino H., Kazama H., Miyazawa K. (2021). Targeted disruption of GAK stagnates autophagic flux by disturbing lysosomal dynamics. Int. J. Mol. Med..

[71] Rontogianni S., Iskit S., van Doorn S., Peeper D.S., Altelaar M. (2020). Combined EGFR and ROCK inhibition in triple-negative breast cancer leads to cell death via impaired autophagic flux. Mol. Cell Proteom..

[72] Huynh K.K., Kay J.G., Stow J.L., Grinstein S. (2007). Fusion, fission, and secretion during phagocytosis. Physiology.

[73] Rojas J., Arraiz N., Aguirre M., Velasco M., Bermúdez V. (2011). AMPK as target for intervention in childhood and adolescent obesity. J. Obes..

[74] Zhou J., Massey S., Story D., Li L. (2018). Metformin: An old drug with new applications. Int. J. Mol. Sci..

[75] Zhou G., Myers R., Li Y., Chen Y., Shen X., Fenyk-Melody J., Wu M., Ventre J., Doebber T., Fujii N. (2001). Role of AMP-activated protein kinase in mechanism of metformin action. J. Clin. Investig..

[76] Park H.W., Park H., Semple I.A., Jang I., Ro S.H., Kim M., Cazares V.A., Stuenkel E.L., Kim J.J., Kim J.S. (2014). Pharmacological correction of obesity-induced autophagy arrest using calcium channel blockers. Nat. Commun..

